# Incidence of Cytomegalovirus (CMV) Infection in After Kidney Transplant Patients: A Systematic Review and Meta‐Analysis

**DOI:** 10.1002/rmv.70092

**Published:** 2026-01-03

**Authors:** Gleice K. Jesus, Róger Costa, Gabriel O. Franco, Cleyde C. S. Marconi, Maria B. Arriaga, Eduardo M. Netto

**Affiliations:** ^1^ Postgraduate Program in Medicine and Health Federal University of Bahia Salvador Brazil; ^2^ Fundação Bahiana de Infectologia Salvador Brazil

**Keywords:** cytomegalovirus, incidence, kidney transplantation, laboratory predictors, opportunistic infection

## Abstract

Kidney transplantation is recognised by the World Health Organisation as the most effective therapy for end‐stage renal disease, offering substantial improvements in survival and quality of life. However, the immunosuppression required to prevent graft rejection predisposes recipients to opportunistic infections, among which cytomegalovirus (CMV) remains a leading cause of morbidity and mortality. To determine the incidence of CMV infection in kidney transplant recipients and identify clinical and laboratory predictors and associated risk factors. A systematic review and meta‐analysis, registered in PROSPERO (CRD42024524165), was conducted in accordance with PRISMA guidelines. PubMed, Web of Science, and LILACS databases were searched up to 6 March 2024 for cohort studies reporting CMV incidence post‐kidney transplantation. Methodological quality was assessed using the Newcastle–Ottawa Scale (NOS). Publication bias was evaluated using funnel plots, Egger's test, Kendall's tau, and the fail‐safe N. Fifteen studies met the inclusion criteria, comprising 3888 patients with a mean age of 45.1 years and a mean follow‐up of 7.14 months. The pooled incidence of CMV infection was 42% (95% CI: 30%–54%), with high heterogeneity (I^2^ = 98.75%; *p* < 0.001). Sensitivity analysis confirmed the robustness of the results, and no significant publication bias was detected. The main risk factors were: advanced age, paediatric age (< 5 years), donor–recipient serodiscordance (D+/R−), post‐transplant lymphopenia, and reduced cell‐specific immunity defect. CMV infection remains a common and clinically significant complication following kidney transplantation. With a high incidence and strong association with specific laboratory and clinical predictors. Individualised prevention strategies and early virological and immunological monitoring, especially in high‐risk groups, are essential to reduce morbidity and mortality and preserve graft function.

**Protocol Registration:**

PROSPERO: CRD42024524165

## 1 Introduction

Kidney transplantation is recognised by the World Health Organisation (WHO) as the most effective therapeutic option for patients with end‐stage renal disease, significantly improving quality of life and increasing survival rates to 83% compared with dialysis [1]. Despite these benefits, the immunosuppression required to prevent graft rejection markedly increases susceptibility to opportunistic infections, affecting up to 57.1% of recipients within 6 months post‐transplant. Among these, cytomegalovirus (CMV) infection is a leading cause of morbidity and mortality, with an overall incidence of 8.8% between the third and sixth months after transplantation [2] and a ninefold higher risk of graft rejection (odds ratio [OR] = 8.9; 95% CI: 2.8–28.1; *p* = 0.001) [3]. Incidence rates vary between populations: 52% in children [4], 60.7% in older adults [5], and up to 75% in donor–recipient serodiscordance (D+/R−) cases [6], the latter representing one of the main risk factors for primary infection and symptomatic disease.

Over 3 decades, the incorporation of sensitive laboratory methods has changed the way CMV is detected after kidney transplantation. The diagnosis of infection can be carried out by serological methods (IgG, IgM), pp65 antigenemia assays, viral culture, or molecular techniques [7]. Quantitative polymerase chain reaction (qPCR) is particularly valuable for early detection of viral replication [4], enabling timely therapeutic interventions and reducing progression to symptomatic disease [8]. It offers superior sensitivity and specificity compared with pp65 antigenemia [1, 2, 5], particularly in leukopenic patients [4, 7].

Cytomegalovirus (CMV) infection is one of the most significant infectious complications in the post‐kidney transplant period, associated with high morbidity and mortality rates and an increased risk of graft rejection. Previous studies indicate incidences ranging from 8.8% to over 70%, with significant variations according to sociological, clinical, and laboratory factors. This variability makes it difficult to define accurate and universal estimates, in addition to limiting the implementation of prevention and monitoring protocols that are appropriate for all clinical contexts.

Unlike syntheses focused on immunosuppressive regimens, this review anchors the incidence of CMV in laboratory markers, emphasising comparable early detection and risk stratification across centres.

This, the present systematic review and meta‐analysis aims to estimate the incidence of CMV infection in kidney transplant recipients, synthesise evidence based on laboratory markers that allow detection, in addition to showing the evolution of diagnostic methods and the usefulness of infection predictors to anticipate events, and finally, identify associated risk factors. An integrated understanding of these metrics can guide more effective preventive and preemptive surveillance strategies, with the potential to reduce clinical complications, preserve graft function, and optimise the prognosis of transplant patients.

## 2 Methodology

### 2.1 Protocol Registration

This systematic review was registered in the International Prospective Register of Systematic Reviews (PROSPERO) [9] under registration number CRD42024524165 (https://www.crd.york.ac.uk/PROSPERO/view/CRD42024524165). The review was conducted following the Preferred Reporting Items for Systematic Reviews and Meta‐Analyses (PRISMA) guidelines [10].

### 2.2 Search Strategy

Searches were performed in PubMed, Web of Science, and LILACS up to March 2024, with filters for English, Spanish, and Portuguese languages and full‐text availability. The following descriptors were used: ‘Cytomegalovirus Infections’ [Mesh] OR ‘cytomegalovirus’ [Mesh] AND ‘Kidney Transplantation’ [Mesh] AND ‘Incidence.’ Equivalent strategies were applied across all databases. See supplement.

### 2.3 Eligibility Criteria

After screening the articles found through the search strategy, those that adequately met the inclusion and exclusion criteria were selected. For inclusion, cohort and case‐control studies published up to March 2024 that evaluated CMV infection in living patients after kidney transplantation, presenting data on the incidence of infection, were selected. Systematic reviews, meta‐analyses, letters to the editor, monographs, studies on infection by other viruses, transplantation of other organs, comparative incidence of medications, and studies without full text available were excluded.

### 2.4 Study Selection

Articles were imported into Rayyan [11] for screening. After duplicate removal, three reviewers (G.J., R.C., C.M.) independently assessed titles and abstracts. Discrepancies were resolved through consensus meetings. In cases where an agreement could not be reached, a fourth reviewer acted as a tie‐breaking mediator. Studies that met the criteria were read in full to confirm eligibility.

### 2.5 Data Extraction

Four authors (G.J., R.C., C.M., G.F.) extracted data into an Excel spreadsheet, including: author, publication year, title, country, study design, sample size, mean age, population, positive cases, follow‐up duration, incidence, time to infection onset, risk factors, diagnostic methods, laboratory variables and conclusions. The data were then included in a Excel worksheet.

### 2.6 Risk of Bias Assessment

The methodological quality of the included observational studies was assessed using the Newcastle‐Ottawa Scale (NOS) [12] tool, as recommended for cohort and case‐control studies. The instrument evaluates three parameters: selection, comparability and outcome/exposure, as can be seen in Table 1. The evaluation was made by one author (G.J.) and verified by two others (G.F. *e* M.A.). Risk of bias was then categorised as high, moderate, or low. The researchers resolved any discrepancies by jointly re‐evaluating a paper.

**TABLE 1:** Evaluation of the methodological quality of studies (NOS).

Author	Selection (max. 4)				Comparability (max. 2)	Exposure (max. 3)			Total
	1	2	3	4	1	1	2	3	
Boland (1990) [13]	*	*	*	*	**	*	*	—	8/9
Pouteil (1992) [14]	*	*	*	*	**	*	—	*	8/9
Toyoda (1997) [15]	*	*		*	**	*	*		7/9
Costa (1999) [16]	*	*	*	*	*	*	*	*	8/9
Alakulppi (2006) [17]	*	*		*	**	*	*		7/9
Cervera (2007) [18]	*	*	*	*	**	*	*		8/9
Sagedal (2008) [6]	*	*	*	*	**	*	*	*	9/9
Salazar (2009) [4]	*	*		*	**	*	*		7/9
Sousa (2010) [2]	*		*	*	**		*	*	7/9
Watcharanan (2012) [19]	*	*		*	**	*	*	*	8/9
Feng (2016) [20]	*	*		*	**	*	*		7/9
Fernández (2019) [3]	*		*	*	**	*		*	7/9
Hemmersbach (2019) [5]	*		*	*	**	*		*	7/9
Sousa (2021) [1]	*	*	*	*	**	*	*	*	9/9
Shiina (2023) [21]	*			*	**	*	*		6/9

### 2.7 Statistical Analysis

For the data analysis of this meta‐analysis, the Jamovi Project programme (Jamovi version 2.4.8) was used. The incidence of CMV infection and their respective 95% confidence intervals (CI) were used as the ratio between the number of positive patients and the total number of patients included in the study. The incidence was expressed as a percentage. Heterogeneity between studies was assessed using the I^2^ statistic. Publication bias was assessed using the funnel plot, Egger's test, and Fail‐Safe N.

## 3 Results

### 3.1 Search Strategy

**FIGURE 1:**
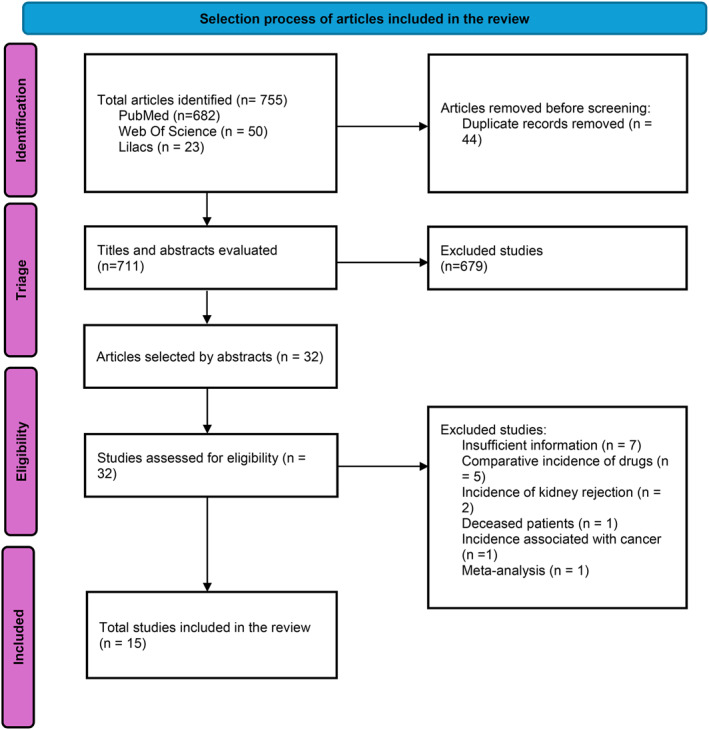
PRISMA flowchart.

A total of 755 potentially eligible articles were identified for inclusion in this review. After removing duplicates (*n* = 44), 711 articles were screened by title and abstract using Rayyan [10]. Of these, 679 articles were excluded, and the remaining 32 were selected for full‐text reading. Subsequently, 17 studies were excluded: 7 for not providing sufficient incidence data, 5 for presenting comparative medication incidence, 2 for addressing kidney graft rejection incidence, 1 for reporting incidence in deceased patients, 1 for associating incidence with cancer, and 1 for being a meta‐analysis. After completing the eligibility assessment, 15 articles were included in the systematic review and meta‐analysis, as shown in Figure 1.

Table 2 of the general characteristics of the studies shows the sample size (*N*) of each study, the total of them was 3888 (median of 159), the mean age was 45.1 and the mean follow‐up time was 7.14 months.

**TABLE 2:** General characteristics of the included studies.

Author	Country	Type of study	Population	*N*	*N* positives	Middle ages	Follow‐up time (months)	Incidence	Diagnosis	Risk factors
Boland [13]	Netherlands	Cohort	Adults	55	25	NR	1–6	0.45	Antigenemia.	Positive IgM
Thumb [14]	France	Cohort	Adults	242	157	NR	1–3	0.65	Antigenemia, culture and serology	Viraemia associated with severe infection
Toyoda [15]	United States	Cohort	Adults	25	9	NR	1–12	0.36	Serology, elisa	High levels of AECA and IL‐2
Coast [16]	Brazil	Cohort		37	32	NR	1–4	0.86	PCR, elisa	NR
Alakulppi [17]	Finland	Cohort	Adults	71	24	47	1–12	0.34	Genotyping, PCR	IL10 genotype
Cervera [18]	Spain	Cohort	Adults	222	39	50	NO^a^	0.18	Genotyping, PCR	Low MBL production genotypes and TLR4 mutation
Sagedal [6]	Norway	Cohort	Adults	159	99	47	1–3	0.62	PCR, MBL and MASP‐2 immunological assays	Low MASP‐2 levels
Salazar [4]	Chile	Cohort	Children	44	14	5.5	1–6	0.32	Indirect immunofluorescence	Previous seronegativity, younger age
Sousa [2]	Brazil	Cohort	Adults	1676	225	41.4	1–12	0.13	PCR and antigenemia	Ischaemia time
Watcharananan [19]	Thailand	Cohort	Adults	218	36	43.3	3–6	0.17	PCR	Higher viral load, acute rejection
Feng [20]	China	Cohort	Adults	319	28	45	1–4	0.09	CMV‐QNAT, elisa, antigenemia	History of rejection, negative expression of anti‐CMV IgG
Fernández‐Ruiz [3]	Spain	Cohort	Adults	124	59	59.8	12	0.48	Cellular immunity monitoring	Low CD8+ count
Hemmersbach‐Miller [5]	United States	Cohort	Elderly	182	118	57.5	12	0.65	Antigenemia	Advancing age and genitourinary conditions
Sousa [1]	Brazil	Cohort	Adults	466	327	52	24	0.70	PCR	Previous seronegativity, absence of prophylaxis
Shiina [21]	Japan	Cohort	Adults	48	13	47	12	0.27	Absolute lymphocyte count	Low lymphocytes count on day 28 post‐day

^a^ NR, not reported.

### 3.2 Risk Bias

To assess the methodological quality and risk of bias of the observational studies included in this systematic review, the Newcastle‐Ottawa Scale (NOS) [12] was used, and all 15 articles included were cohort articles and were endorsed by the NOS cohort model. The scale assigned up to 9 points distributed among the parameters.

In the Table 1, the studies evaluated obtained scores ranging from 6 to 9 points.

**FIGURE 2:**
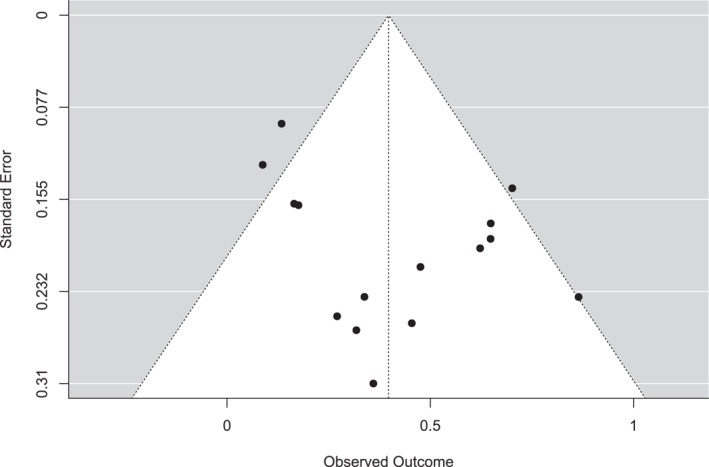
Funnel plot for publication bias assessment. Funnel plot showing publication bias evaluation among the meta‐analysis included studies.

The presence of publication bias was also assessed using the funnel plot, as shown in Figure 2, in addition to the Fail‐Safe *N* = 11.326 (*p* < 0.001), Kendalls Tau = 0.067 (*p* = 0.770) and Egger's Regression = 0.633 (*p* = 0.527) tests. These results confirm that there is no evidence of publication bias, demonstrating the stability and reliability of this meta‐analysis.

The funnel plot analysis was complemented by Kendall's Tau (value 0.124), Egger's regression (value 1.394), and Fail‐Safe *N* (363.000) tests. And despite the slight asymmetry in the funnel plot, none of the complementary tests showed significant evidence of publication bias, and the high Fail‐Safe N suggests that the results of the meta‐analysis are robust.

### 3.3 Statistical Analysis

The present meta‐analysis, consisting of 15 studies, identified a pooled incidence of CMV infection of 0.42 (95CI: 0.30%–0.54%), considering follow‐up periods ranging from 1 to 24 months. These results demonstrate a robust and clinically relevant finding. The I^2^ value was 98.75% (*p* < 0.001), showing high heterogeneity among the studies. Therefore, the meta‐analysis was carried out under a random‐effects model.

**FIGURE 3:**
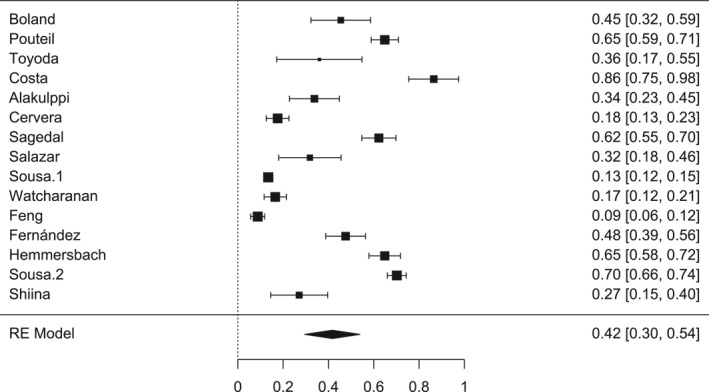
Forest plot. Forest plot of the studies included in the meta‐analysis. Each line represents the point estimate and 95% confidence interval of each study. The black diamond represents the combined effect according to the random effects model, with an estimated value of 0.42 [0.30, 0.54].

Figure 3 presents the individual results of the studies included in the meta‐analysis, as well as the estimated pooled effect. It is observed that, although some studies present wider confidence intervals or results that are not statistically significant in isolation, such as Toyoda et al. [15], Salazar et al. [4] and Shiina et al. [21], most of them demonstrate a consistent association with the occurrence of CMV infection in different clinical and population contexts.

The estimate evidenced reflects the aggregate prevalence of infection among the studies, considering methodological and sample variations. The studies with the greatest weight in the analysis, such as Sousa et al. [1], Pouteil‐Noble et al. [14], Hemmersbach‐Miller et al. [5] corroborate this finding, reinforcing the consistency of the data and the reliability of the results.

Despite the heterogeneity between the studies, possibly due to differences in diagnostic methods, populations evaluated and follow‐up periods, as demonstrated by Salazar et al. [4], Sousa et al. [1, 2] and Feng et al. [20], the findings support the clinical relevance of CMV infection in the context of kidney transplantation. The body of evidence supports the importance of virological monitoring and the implementation of appropriate preventive strategies, especially in higher‐risk groups, such as patients with D+/R serology–, children, and the elderly.

Also in Figure 3, it is observed that two works by the same author, Sousa et al. [1] and Sousa et al. [2], carried out in different years, presented very different incidences of CMV. The 2010 study recorded an incidence of 0.13 (95 CI: 11%–15%), while the 2021 study had a significantly higher incidence of 0.70 (95 CI: 66%–74%). A possible explanation for this discrepancy is the age profile of the populations analysed: in 2021, the authors included older patients (mean of 52 years), while in 2010 they evaluated younger patients (mean of 41 years). Considering that advanced age is a recognized risk factor for CMV infection, this demographic difference may have had a direct influence on the higher incidence observed in Sousa's study in 2021 [2].

### 3.4 Laboratory Predictors and Diagnostic Performance

In classic and contemporary series, qPCR detected CMV before serology, including as the only positive method in some cases, supporting its use as an early detection tool [16]. The pp65 antigenemia appeared, on average, on day 45. Cellular specific anti‐CMV immunity measured early predicted subsequent events, with a higher incidence of infection in patients with no or weak response [3]. ALC‐D28 (absolute lymphocyte count 28th day) < 1100/μL showed useful operational PPV (83% NPV) and a 3.3‐fold higher risk of CMV in the 1st year [21].

Positive IgM and viruria were associated with moderate/severe forms (ratio of 3.3 for severity with positive IgM) [14]. Low levels of MASP‐2 in the pre‐transplant period were associated with the development of CMV disease in the first 12 weeks (*p* = 0.028) [6]. In parallel, AECA (Anti‐endothelial antibodies) rose 1–4 weeks after detection of CMV DNA and remained high for months, suggesting endothelial injury and possible impact on rejection [15].

Some studies presented risk factors or reduction factors for infection among the patients studied, as shown in Table 1. Alakulppi et al. [17] observed a lower incidence when the donor has the IL‐10 genotype (−1082 AA), with a *p*‐value = 0.036. Feng et al. [20], showed more variations in risk factors.

The synthesis of clinical and laboratory predictors (Table 1) highlights important characteristics of early detection. qPCR identified subclinical infection before antigenemia, including as the only positive method in some cases [16]. Shiina et al. [21] It was observed that the absolute lymphocyte count on the 28th post‐transplant day (ALC‐D28 < 1100/μL) was associated with a higher incidence of CMV (HR = 3.32; VPN = 83%). In the study by Fernandéz‐Ruiz et al. [3] it was seen that reduced anti‐CMV specific cellular responses, assessed in the pre‐ and post‐immediate, predicted subsequent CMV events. And components of the complement lectin pathway, especially MASP‐2, have been associated with early CMV disease [6]. Together, these markers allow for reproducible risk stratification and guide surveillance and preemptive intervention (Table 3).

**TABLE 3:** Clinical and laboratory predictors.

Study	Preditor	Incidence	Observations
Boland [13]	pp65 antigenemia	HIV‐positive with active infection: 74%	Antigenemia precedes IgG/culture; useful for pre‐emptive intervention
	Serodiscordance D+/R−	Primary infection: 36%	Highest risk group for primary CMV
Pouteil‐Noble [14]	Positive IgM/viruria	65%	Association with moderate/severe forms (OR ≈ 3.28)
Toyoda [15]	Elevated post‐CMC‐DNA AEC	NR	Elevated AECA 1–4 weeks after CMV‐DNA; they are maintained for months; possible vascular rejection
Coast [16]	qPCR (blood/urine)	Active infection (any method): 86.4%; urine CRP: 64.9%	PCR detects before serology; some PCR‐positive cases only
Alakulppi [17]	Donor IL‐10 genotype (−1082 AA vs. AG/GG)	15% (AA) and 41% (AG/GG)	Associated polymorphism; scenario D+/R−; prophylaxis varied among patients
Cervera [18]	TLR4 polymorphism	58.4%	Presence of the mutation increases the risk of primary CMV
Sagedal [6]	MASP‐2 low (≤ 148 μg/L)	CMV disease at 12 weeks: 22%; overall infection: 59.8%	No prophylaxis; biweekly PP65 tracking
Salazar [4]	Paediatric (< 5 years)	Up to 6 months: 45%; condition: 11%	Greater precocity of events in paediatrics
Watcharananan [19]	Prior acute rejection/high viral load (qPCR)	4.6%	Approximate cut‐off point: 1.7 × 10^4 copies/mL versus. < 7.2 × 10^3
Feng [20]	Leucopenia and high viral load	8.8%	Leucopenia favours viral infections
	Anti‐CMV IgG positive	9%–12%	Combined with other factors
	Advanced age (≥ 65 years)	60.7%	Increased vulnerability
	Post‐transplant lymphopenia	44%	Patient with lymphopenia on the 28th day developed CMV within 1 year
	Pre‐transplanted MIC deficiency	71%	39% when CD8^+^ ≥ 1 cél/mL
Fernández‐Ruiz [3]	Absent/weak CMI (specific T‐response)	At 12m: 47.6% (viraemia 39.5%)	Reduced cellular response in the pre/post‐immediate predicts events
Sousa—2 [1]	Diabetes mellitus	73.7%	Associated with other infections, mainly by multidrug‐resistant microorganisms.
Shiina [21]	ALC‐D28 < 1.100/μL	27% (stratified by LAC: NR)	HR 3.32 for low LAC; VPN 83% to the cutoff point

Abbreviations: ALC‐D28, absolute lymphocyte count on day 28; MIC AECA, anti‐endothelial antibodies; NR, not reported; qPCR, quantitative PCR.

## 4 Discussion

Our findings reinforce that surveillance strategies anchored in laboratory predictors allow us to estimate the incidence and anticipate the risk of CMV infection in a comparable manner. Older studies, based on serology or antigenemia, probably underestimated the incidence and detected the events later [13, 14]. On the other hand, the progressive incorporation of molecular methods such as qPCR, since the end of the 90s [16] and monitoring of specific cellular immunity has shifted the identification of cases to earlier stages of the post‐transplant period and revealed subclinical infection. This methodological evolution explains part of the heterogeneity observed and, at the same time, justifies the inclusion of historical series to understand the trajectory of the disease over almost 3 decades [3, 21] (Table 4).

**TABLE 4:** Laboratory markers and clinical implications for CMV infection.

Study	Risk factors	Prevention or monitoring strategy
Boland [13]	Serodiscordance D+/R−: Primary infection 36%; active 74% (seropositive)	Early surveillance with antigenemia; preemptive intervention when positive
Pouteil‐Noble [14]	Positive IgM: High severity (OR ≈ 3.28)	Combine IgM + viruria/viraemia to stratify severity and anticipate therapy
Toyoda [15]	Elevated AECA: Suggests associated vascular risk/rejection	Monitor signs of endothelial injury; consider impact on surveillance and rejection
Coast [16]	Early detection by qPCR: Identifies initial subclinical infection	Implement serial qPCR as the basis of pre‐emptive protocol
Alakulppi [17]	Donor IL‐10 genotype (−1082 AA vs. AG/GG)	Consider genotyping as risk refinement
Cervera [18]	Induction with ATG; D+/R− risk: Increased risk with lymphodepletion	Prophylaxis with ganciclovir; PP65‐guided preemptive;
Sagedal [6]	Low MASP‐2/MBL	Intensified surveillance without prophylaxis
Salazar [4]	Paediatric range	Intensive monitoring in the first months; lower thresholds for intervention
Sousa—1 [2]	Early infectious complications: CMV as one of the main infections	Apply systematic laboratory screening protocols
Watcharanan [19]	Previous acute rejection; high viral load by qPCR: Association with symptomatic CMV	Use viral load cut‐off points to initiate pre‐emptive therapy; heightened post‐rejection surveillance
Feng [20]	D+/R−; IgG negative pre‐transplant; leucopenia/high viral load: high risk in subgroups	Stratify by serology; reinforce prophylaxis and serial qPCR at high risk
Fernández‐Ruiz [3]	Reduced IMR: Higher incidence of events at 12 months	Monitor CMI in the pre‐ and post‐immediate; modular qPCR frequency/prophylaxis
Hemmersbach‐Miller [5]	Elderly (≥ 65 years old) Elevated risk of infections in year 1	Enhanced surveillance protocols; integration with qPCR and clinical assessment
Sousa—2 [1]	DECD: CMV as the most common infection; independent association with MDR	Intensified surveillance and antimicrobial stewardship; attention to MDR
Shiina [21]	ALC‐D28 < 1.100/μL HR 3.32; VPN 83% for cutoff point	Use ALC‐D28 as screening for monitoring density in the 1st year

Abbreviations: AECA = anti‐endothelial antibodies; ALC‐D28 = absolute lymphocyte count on day 28; ATG = antithymocyte globulin; DECD = deceased donor with expanded criteria; HR = hazard ratio; IL‐10 = interleukin‐10; MBL/MASP‐2 = components of the lectin pathway; MDR = multidrug‐resistant; NPV = negative predictive value; pp65 = antigenemia; qPCR = quantitative PCR.

Based on the studies analysed, CMV infection continues to be a frequent event after kidney transplantation with a wide variation in incidence, according to diagnostic method, population profile, and follow‐up window. The study by Sousa et al. [2], reported an incidence of 8.8% between the third‐ and sixth‐months post‐transplant, while Feng et al. [20], found rates of 47% among recipients with unfavourable immunological and serological risk factors. Despite its high frequency, studies suggest that when identified and treated early and treated appropriately, CMV infection does not compromise the survival of the actual graft. When the infection is not controlled, it can cause acute rejection, viral nephropathy, and graft failure [1]. Shiina et al. [21] showed that patients with low cellular immunity had CMV infection of more worrisome clinical condition (HR = 3.32; 95% CI = 1.08–10.2).

Stratification by subgroups reinforced the clinical applicability of the findings. Children and the elderly had a higher incidence and severity of events [4, 5]. D+/R− serodiscordance remained the main risk factor for primary infection and disease [14]. In recipients of donors with expanded criteria, CMV was the most common complication, independently associated with infection by multidrug‐resistant microorganisms [1]. Such patterns not only reduce heterogeneity through more homogeneous comparisons, but also enrich the clinical relevance of the review by guiding who to monitor more closely, when, and with which tools.

The absence of direct analysis by immunosuppressive regimens can be understood as a limitation, but it was a conscious methodological option to avoid confusion due to the great variation of regimens in studies. Instead, we emphasise laboratory markers that are comparable over time and useful for preemptive treatments: serial qPCR as the screening axis [16], pp65 [13] and ALC‐D28 [21] to qualify initial risk, specific cellular immunity to modulate intensity [3] or duration of surveillance, and IgM/viruria as severity alarms [20].

In summary, by centring incidence on laboratory predictors and arranging markers in post‐transplant timelines, this review explains historical heterogeneity, standardises comparison between studies, and delivers actionable parameters for pre‐emptive surveillance and risk stratification in post‐kidney transplant care.

## 5 Conclusion

This review confirms that CMV infection remains one of the most frequent and clinically relevant infectious complications in kidney transplant recipients, presenting mainly in the first few months after the procedure. The estimated pooled incidence was 42% (95% CI: 30%–54%), with high heterogeneity (I^2^ = 98.75%), reflecting substantial differences between studies in terms of population profiles, immunosuppressive regimens employed, diagnostic methods, and prophylaxis strategies adopted.

By refocusing the analysis on laboratory markers, this work offers a practical and standardisable path for surveillance: serial qPCR as the basis of early detection, pp65 antigenemia as the typical operational window. ALC‐D28 < 1100/μL as a simple screening point to intensify follow‐up, MIC to modulate the frequency and duration of monitoring, and, as complementary signs, low MASP‐2 pre‐transplant and elevated AECA after viraemia, suggesting greater susceptibility and possible vascular impact.

This approach is independent of the variability of immunosuppressive regimens between centres and eras and speaks directly to the care routine: it allows for earlier action, personalisation of risk, and protection of the graft. Sensitivity analysis in modern scenarios where qPCR is the gold standard reinforces the robustness of these conclusions.

## Author Contributions

The conceptualisation of the study was carried out by Gleice K. Jesus and Eduardo M. Netto. Data curation was conducted by Gleice K. Jesus, Róger J. Costa, Gabriel O. Franco and Cleyde C. S. Marconi. The formal analysis had the participation of Gleice K. Jesus, Gabriel O. Franco and Eduardo M. Netto, while the methodology was developed by Gleice K. Jesus, Róger J. Costa, Cleyde C. S. Marconi and Eduardo M. Netto. The project was managed by Gleice K. Jesus and Eduardo M. Netto. Statistical analysis was performed by Gleice K. Jesus, Gabriel O. Franco, Maria B. Arriaga and Eduardo M. Netto. The orientation was the responsibility of Eduardo M. Netto, and the validation was carried out by Gleice K. Jesus and Eduardo M. Netto. The original draft was written by Gleice K. Jesus, and all authors participated in the writing, revision, and editing, having access to all the data, contributing, revising, and approving the final version of this manuscript, as well as assuming final responsibility for the decision to submit it for publication.

## Funding

GJ (166623/2023‐7) and CM (140187/2021‐9) are recipients of a Brazilian National Research Council (CNPq) scholarship; GF (88887.960010/2024‐00) and RC (8887.91553/2023‐00) are recipient of a Coordination for the Improvement of Higher Education Personnel scholarship (CAPES); the project received funding from CNPq (404193/2019.6). The funders were not involved in the conceptualisation, data extraction, analysis, preparation of the manuscript and the decision to publish.

## Ethics Statement

The authors have nothing to report.

## Consent

The authors have nothing to report.

## Conflicts of Interest

The authors declare no conflicts of interest.

## Supporting information


Supporting Information S1

Supporting Information S2

Supporting Information S3

## Data Availability

All data, extraction forms, and analysis codes are available upon request to the corresponding author.
