# Supplementary material for: Incidence of Cytomegalovirus (CMV) Infection in After Kidney Transplant Patients: A Systematic Review and Meta‐Analysis
Source: Rev Med Virol. 2026 Jan 3;36(1):e70092. doi: 10.1002/rmv.70092 (PMC12764368; doi:10.1002/rmv.70092)
Supplement: Supplementary file 1 — Supporting Information S1 [file RMV-36-e70092-s001.docx]

WHO – World Health Organization

CMV – Cytomegalovirus

OR – Odds Ratio

CI – Confidence Interval

D+/R− – Donor positive/Recipient negative

IgG – Immunoglobulin G

IgM – Immunoglobulin M

PCR – Polymerase Charin Reaction

qPCR – Quantitative Polymerase Chain Reaction

pp65 – pp65 antigenemia assay

ALC – Absolute lymphocyte Count

PROSPERO – International Prospective Register of Systematic Reviews

PRISMA – Preferred Reporting Items for Systematic Reviews and Meta-Analyses

NOS – Newcastle-Ottawa Scale

NR – Not reported

MBL – Mannose-Binding Lectin

TLR4 – Toll-Like Receptor 4

MASP-2 – Mannan-binding lectin serine protease 2

AECA – Anti-Endothelial Cell Antibodies

IL-2 – Interleukin-2

IL-10 – Interleukin-10

IL-10 – 1082 A/G - Promoter polymorphism at position −1082 (alleles A or G) in the *IL10* gene

AG/GG (G-allele carriers) — Grouping of G-allele carriers (AG or GG) at IL10 −1082 A/G

HR – Hazard Ratio

ICU – Intensive Care Unit

ATG – Anti-Thymocyte Globulin

ICS – Intracellular Cytokine Staining

CMI — CMV-specific Cellular Immunity

DECD — Deceased Expanded Criteria Donor

NPV — Negative Predictive Value

PPV — Positive Predictive Value

D15 / D28 — Day 15 / Day 28 (post-transplant)

I² — I-squared heterogeneity statistic
