# Supplementary material for: Incidence of Cytomegalovirus (CMV) Infection in After Kidney Transplant Patients: A Systematic Review and Meta‐Analysis
Source: Rev Med Virol. 2026 Jan 3;36(1):e70092. doi: 10.1002/rmv.70092 (PMC12764368; doi:10.1002/rmv.70092)
Supplement: Supplementary file 2 — Supporting Information S2 [file RMV-36-e70092-s002.docx]

S1 Appendix

**Search strategy (PubMed)**

("Cytomegalovirus Infections" [Mesh] OR "cytomegalovirus" [Mesh] AND "Kidney Transplantation" [Mesh] AND "Incidence")

**Search strategy (Web of Science)**

(ALL= (Cytomegalovirus Infections) OR ALL=(cytomegalovirus) AND (ALL=Kidney Transplantation) AND (ALL=Incidence))

**Search strategy (LILACS)**

((cytomegalovirus infections) OR (cytomegalovirus) AND (kidney transplantation) AND (incidence))
